# Effect of a calcium hydroxide-based intracanal medicament containing *N*-2-methyl pyrrolidone as a vehicle against *Enterococcus faecalis* biofilm

**DOI:** 10.1590/1678-7757-2019-0516

**Published:** 2020-03-27

**Authors:** Taegun KIM, Mi-Ah KIM, Yun-Chan HWANG, Vinicius ROSA, Massimo DEL FABBRO, Kyung-San MIN

**Affiliations:** 1 Chonbuk National University School of Dentistry Institute of Oral Bioscience Jeonju Korea Chonbuk National University, School of Dentistry and Institute of Oral Bioscience, Department of Conservative Dentistry, Jeonju, Korea.; 2 Chonnam National University School of Dentistry Department of Conservative Dentistry Gwangju Korea Chonnam National University, School of Dentistry, Department of Conservative Dentistry, Gwangju, Korea.; 3 National University of Singapore Faculty of Dentistry Singapore National University of Singapore, Faculty of Dentistry, Discipline of Oral Sciences, Singapore.; 4 Università degli Studi di Milano Dipartimento di Scienze Biomediche, Chirurgiche e Odontoiatriche Milano Italia Università degli Studi di Milano, Dipartimento di Scienze Biomediche, Chirurgiche e Odontoiatriche, Milano, Italia.; 5 IRCCS Istituto Ortopedico Galeazzi Milano Italia IRCCS Istituto Ortopedico Galeazzi, Milano, Italia.; 6 Chonbuk National University Research Institute of Clinical Medicine Jeonju Korea Chonbuk National University, Research Institute of Clinical Medicine, Jeonju, Korea.; 7 Chonbuk National University Hospital Biomedical Research Institute Jeonju Korea Chonbuk National University Hospital, Biomedical Research Institute, Jeonju, Korea.

**Keywords:** Biofilms, Enterococcus faecalis, Calcium hydroxide

## Abstract

This study investigated the effect of a calcium hydroxide (CH) paste (CleaniCal^®^) containing *N*-2-methyl pyrrolidone (NMP) as a vehicle on *Enterococcus faecalis* (*E. faecalis*) biofilms compared with other products containing saline (Calasept Plus™) or propylene glycol (PG) (Calcipex II^®^).

**Methodology:**

Standardized bovine root canal specimens were used. The antibacterial effects were measured by colony-forming unit counting. The thickness of bacterial microcolonies and exopolysaccharides was assessed using confocal laser scanning microscopy. Morphological features of the biofilms were observed using field-emission scanning electron microscopy (FE-SEM). Bovine tooth blocks covered with nail polish were immersed into the vehicles and dispelling was observed. The data were analyzed using one-way analysis of variance and Tukey tests (p<0.05).

**Results:**

CleaniCal^®^ showed the highest antibacterial activity, followed by Calcipex II^®^ (p<0.05). Moreover, NMP showed a higher antibacterial effect compared with PG (p<0.05). The thickness of bacteria and EPS in the CleaniCal^®^ group was significantly lower than that of other materials tested (p<0.05). FE-SEM images showed the specimens treated with Calasept Plus™ were covered with biofilms, whereas the specimens treated with other medicaments were not. Notably, the specimen treated with CleaniCal^®^ was cleaner than the one treated with Calcipex II^®^. Furthermore, the nail polish on the bovine tooth block immersed in NMP was completely dispelled.

**Conclusions:**

CleaniCal^®^ performed better than Calasept Plus™ and Calcipex II^®^ in the removal efficacy of *E. faecalis* biofilms. The results suggest the effect might be due to the potent dissolving effect of NMP on organic substances.

## Introduction

*Enterococcus faecalis* (*E. faecalis*) is a gram-positive facultative anaerobe mainly related to failed root canal treatment.^1^ It can resist very harsh environmental conditions by invading dentinal tubules, enduring prolonged periods of starvation, and forming intraradicular biofilms that are more resistant to antimicrobial agents.^2-4^ Among these, the ability to form biofilms is one of the main mechanisms for the survival of bacterial species in the root canal system. Microbes in biofilms are much harder to eradicate using antimicrobial agents compared with corresponding planktonic forms.^5^ In this respect, it is necessary to use intracanal medicaments in interappointment such as calcium hydroxide (CH) for further disinfection in addition to mechanical instrumentation with irrigants during endodontic treatment to reduce microorganisms in the root canal system. However, the disinfection efficacy of CH is hindered in the presence of biofilms;^4,6^ therefore, enhanced efficacy in biofilm removal of intracanal medicaments has been demanded nowadays.

CH has a long history of use as an endodontic intracanal medicament due to its antibacterial effect.^7^ Thus, liquid vehicles are required for CH to facilitate the delivery of dry powder and the release of hydroxyl ions.^8^ The vehicles are also used to improve antibacterial effect and biocompatibility.^9,10^ Basically, three main types of vehicle exist: (i) water-soluble substances including water, saline, and methylcellulose; (ii) viscous vehicles such as glycerin, polyethylene glycol (PEG), and propylene glycol (PG); (iii) oil-based vehicles such as olive oil, silicone oil, and some fatty acids.^8^ If the vehicle *per se* has antibacterial effects on biofilms, it would be more beneficial for the prognosis. However, most vehicles currently used do not have a remarkable antibacterial effect. Recently, a CH-based intracanal medicament containing *N*-2-methyl-pyrrolidone (NMP) as a vehicle (CleaniCal^®^, Maruchi, Wonju, Korea) was developed. NMP is a colorless organic solvent with high boiling point, low viscosity, low toxicity, and good biocompatibility.^11^ The NMP is a very strong solubilizing agent that has important applications in different fields of industry.^12^ To our knowledge, CleaniCal^®^ is the first commercially available CH-based intracanal medicament containing NMP. However, no published evidence regarding its antibacterial effects can be found. Therefore, this study aimed to investigate the effect of a CH-based paste containing NMP as a vehicle on *E. faecalis* biofilms formed in bovine root canals in comparison with other products containing water-soluble (saline) or viscous (PG) vehicles. The null hypothesis was that no difference among the three materials tested in terms of biofilm removal would be found.

## Methodology

### Preparation of specimens

Standardized root canal specimens obtained from freshly extracted single-rooted bovine central incisors were used as described in a previous study.^13^ They were immersed for 24 h in 1% sodium hypochlorite solution for surface disinfection. The teeth were horizontally sectioned below the cementoenamel junction with a diamond saw (AEU-25, Aseptico, Woodinville, WA, USA) at 15,000 rpm to a length of 5 mm. The root canal was enlarged with a 3.1 mm diameter round bur and then vertically sectioned with a diamond saw into cylindrical halves. The smear layer was removed using 17% ethylenediaminetetraacetic acid solution for 1 min. The specimen was sterilized in an autoclave (LAC-5101SD, Daihan Lab Tech, Namyangju, Korea) at 121°C for 20 min to prevent bacterial contamination. The outer surface of the sectioned specimens was varnished with a double layer of nail polish to ensure dentin contamination only through the main root canal wall.

### Colony-forming unit (CFU) counting assay for vehicles

*E. faecalis* (bacterial strain ATCC 29212) was cultured aerobically in sterile brain heart infusion media (BHI; Difco Laboratories, Detroit, MI, USA) at 37°C overnight. BHI plates were prepared with the addition of 1.5% (wt/vol%) agar (Difco Laboratories). Then, *E. faecalis* (6×10^5^ CFU/mL) was transferred to a 96-well plate (SPL, Daejeon, Korea) that contained 20% (v/v%) of saline, PG (Daeheung Chemicals & Metals, Siheung, Korea), and NMP (Sigma-Aldrich, St Louis, MO, USA) with 80% of BHI media. After incubation at 37°C for 24 h, they were serially diluted and plated on BHI agar plates and CFUs of each sample were counted (n=6).

### Biofilm formation in bovine root canal specimen

After the culture of *E. faecalis* in sterile BHI media containing 1.5% (wt/vol%) agar at 37°C overnight, *E. faecalis* (6×10^5^ CFU/mL) was transferred to a 24-well plate (SPL). Then, the bovine root canal specimens were placed vertically and incubated at 37°C for 7 days for biofilm formation.

### CFU counting assay for the intracanal medicaments

The bovine root canal specimens of the experimental groups were filled with the materials tested, including Calasept Plus™(Nordiska Dental AB, Ängelholm, Sweden), Calcipex II^®^ (Nippon Shika Yakuhin, Shimonoseki, Japan), or CleaniCal^®^, and maintained at 37°C for 24 h. The specimens in the control group were infected with *E. faecalis* such as the experimental groups but received no treatment with CH-containing products. Then, the specimens of the experimental groups were irrigated with 5 ml of sterile water and gently sonicated (10 s, twice at 20% energy level) to remove the medicaments using a sonifier (VCX 130PB; Sonics & Materials, Newtown, CT, USA). The six specimens (n=6) were transferred to a 1.5 ml tube containing 1 ml of sterile water. An aliquot (0.1 ml) of each specimen was serially diluted and plated on BHI agar plates and incubated at 37°C. After 24 h, CFUs of each sample were counted.

### Confocal laser scanning microscopy (CLSM) analysis

One μM of Alexa Fluor 647-labeled dextran conjugate (Molecular Probes, Eugene, OR, USA) was added to BHI broth with *E. faecalis* (6×10^5^ CFU/ml) and incubated for 7 days at 37°C. The fluorescence-labeled dextran can be incorporated during the exopolysaccharide (EPS) matrix synthesis over the course of biofilm development. After a 7-days incubation, three specimens were treated with the medicaments tested for 24 h and washed with distilled water (DW) (n=3). Then, the specimens (n=6) were transferred to 1.5 ml test tubes with 1 ml of DW and sonicated for 30 s in a water bath (JS Research Inc., Gongju, Korea) to remove the pastes. The bacteria remained on the specimens were labeled with 2.5 μM SYTO 9 green fluorescent nucleic acid stain (480/500 nm; Molecular Probes) for 30 min at room temperature. CLSM imaging of the biofilms was performed using a LSM 510 META microscope (Carl Zeiss, Jena, Germany). The thickness of bacterial microcolonies and EPS were quantified based on the confocal stacks using COMSTAT (http://www.comstat.dk; Technical University of Denmark, Kongens Lyngby, Denmark).

### Field-emission scanning electron microscopy (FE-SEM) observation

After treatment with the materials tested, the specimens were transferred to 1.5 ml tubes containing 1 ml of DW and sonicated for 30 s in a water bath to remove the pastes. Then, the biofilms remained on the specimens were fixed in 2.5% glutaraldehyde (Sigma-Aldrich, St. Louis, MO, USA) and then dehydrated in a graded series of ethanol (25–100%) and a critical point dryer (Leica EM CPD300, GmbH, Vienna, Austria). The samples were then observed by FE-SEM (Hitachi, Tokyo, Japan).

### Removal effect of nail polish on bovine tooth blocks

The following experiment using nail polish and bovine tooth blocks was performed to verify whether NMP can dispel organic substances from inorganic tooth surfaces. The bovine tooth block (5 mm x 5 mm) was obtained from the crown portion of bovine teeth. Then, red-colored nail polish was varnished on the enamel surface of the block. The blocks were immersed in water, saline, PG, or NMP, respectively. After 2 h, the surface was observed using a stereo microscope (MZ16FA; Leica Microsystems, Wetzlar, Germany) to verify whether the nail polish was dispelled.

### Statistical analysis

The data were statistically analyzed using Kolmogorov-Smirnov test for determination of normal distribution, and one-way analysis of variance (ANOVA) and Tukey tests were used to detect any signiﬁcance. These analyses were performed using the SPSS software (SPSS 12.0 K for Windows; SPSS Inc., Chicago, IL, USA). A *p-*value lower than 0.05 was considered statistically significant.

## Results

### CFU assay

**Figure 1:**
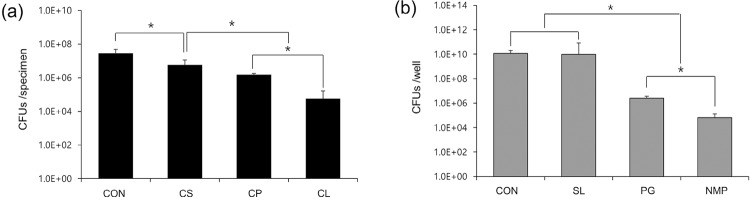
(a and b) CFU counting values of the medicaments tested and the vehicles, respectively. One-way ANOVA and Tukey test were performed. *Statistical significance was determined at p<0.05. CON: control, CS: Calasept Plus™, CP: Calcipex II®, CL: CleaniCal®, SL: saline, PG: propylene glycol, NMP: *N*-2-methyl-pyrrolidone

The CFU assay of *E. faecalis* in biofilms after the intracanal medicament treatments showed all the materials tested had antibacterial effects of 96% (CleaniCal^®^), 82% (Calcipex II^®^), and 70% (Calasept Plus™), respectively (Figure 1a). The groups showed significant difference between them (p<0.05). Furthermore, NMP had the highest antibacterial activity, followed by PG (p<0.05) (Figure 1b).

### CLSM and FE-SEM analysis

**Figure 2:**
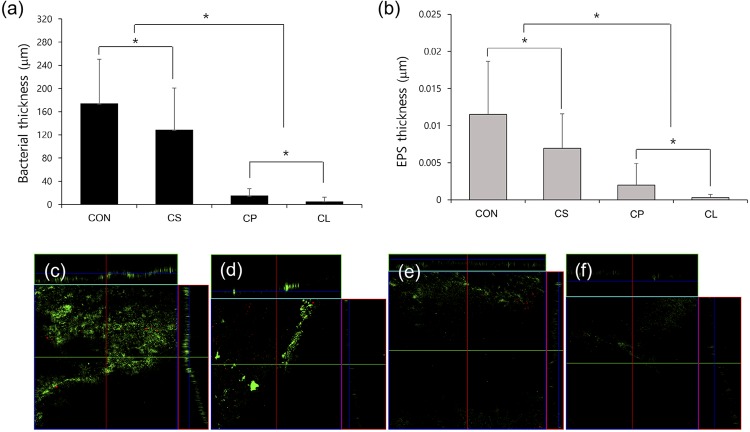
Effect of different medicaments on *E. faecalis* biofilms. (a) Bacterial thickness, (b) EPS thickness, (c-f) representative CLSM images of EPS; (c) Control, (d) Calasept Plus™, (e) Calcipex II®, and (F) CleaniCal®. One-way ANOVA and Tukey test were performed. *Statistical significance was determined at p<0.05. CON: control, CS: Calasept Plus™, CP: Calcipex II®, CL: CleaniCal®

**Figure 3:**
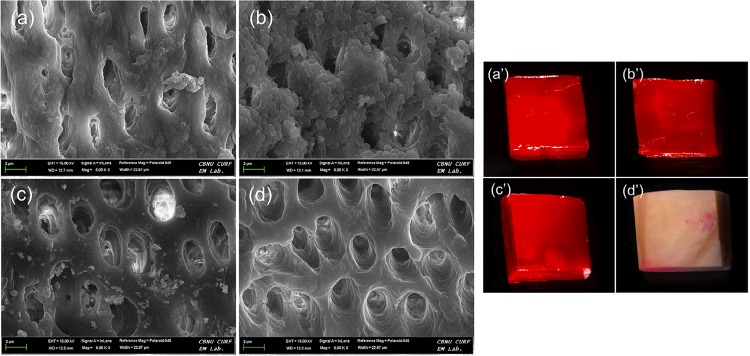
(a-d) Representative FE-SEM images after removal of pastes from *E. faecalis* biofilms formed on the specimens (5,000X). (a’-d’) Stereomicroscopic images of bovine tooth blocks showing remaining nail polish; Control (a and a’), Calasept Plus™ (b and b’), Calcipex II® (c and c’), and CleaniCal® (d and d’)

The CLSM images of the biofilms after the medicament treatments showed bacterial micro-colonies and EPS thickness. As shown in Figure 2, the bacterial and EPS thickness of Calcipex II^®^ and CleaniCal^®^ groups were significantly lower than those of the control and Calasept Plus™ groups (p<0.05). In addition, CleaniCal^®^ showed lower microcolonies and EPS thickness than Calcipex II^®^ (*p*<0.05). FE-SEM images showed that the root canal surfaces of both control and Calasept Plus™-treated group were fully covered by biofilms, whereas the canal surfaces treated with Calcipex II^®^ and CleaniCal^®^ were not (Figure 3a-d). Notably, the specimen treated with CleaniCal^®^ was cleaner than the one treated with Calcipex II^®^. Furthermore, the nail polish on the enamel surface of the bovine tooth block immersed in NMP was completely dispelled after 24 h, whereas the nail polish was not dispelled at all in the other groups (Figure 3a’-d’).

## Discussion

In this study, the effect of CleaniCal^®^ — the NMP-based intracanal medicament — on *E. faecalis* biofilms cultured on bovine root canal walls was investigated comparing it with other products containing different vehicles such as saline (Calasept Plus™) and PG (Calcipex II^®^). Firstly, the effect of the tested materials on biofilms in the root canal was investigated by measuring CFUs. As shown in Figure 1a, all the tested medicaments showed lower CFU values compared with the control. However, CleaniCal showed the lowest value. CH releases hydroxyl ion, which is responsible for the antibacterial activity and dissolution of organic tissues. Therefore, the content of CH in the medicament and hydroxyl ion releasing efficacy are the main key attributes to remove biofilms. According to the manufacturer’s information, the CH content of the products tested are different (Calasept Plus™ ≈ 41%, Calcipex II^®””^ ≈ 24%, CleaniCal^®^ ≈ 30%). Although Calasept Plus™ contains more CH, the antibacterial effect on *E. faecalis* was lower than that of other products that contain less CH. In this respect, the conclusion was that CH content could not explain the CFU results. The ability of releasing hydroxyl ions from CH in a given product is more critical than CH content itself. Furthermore, in general, water-based CH paste releases more hydroxyl ions and raises the mouth pH relative to the other pastes containing viscous vehicles, although controversies remain in the literature.^7,9,10,14,15^ Therefore, it was assumed that the vehicle *per se* might be the factor and the antibacterial effect of each vehicle including saline, PG, and NMP was assessed. In this study NMP showed lower CFU values than PG (Figure 1B). Phaechamud, et al.^16^ (2012) showed NMP has antimicrobial activity against several bacteria. It is known that NMP solubilizes lipids in the cell membrane and promotes the leakage of microbial cell membranes. Therefore, it was considered that the antibacterial property of NMP can partly explain the observed effect of CleaniCal^®^ on biofilms.

Secondly, the *E. faecalis* biofilm structure was characterized by measuring the number of remaining bacterial microcolonies and the amount of remaining EPS using CLSM. EPS is the crucial component of the protective shelter in oral biofilms.^17^ It provides mechanical stability and drug tolerance to biofilms so that the biofilms become resistant to intracanal medication. Therefore, EPS is considered as a new target for antibacterial therapies and measuring the amount of remaining EPS can be a quantitative method for efficacy in biofilm removal. As shown in Figure 2, the biofilms treated with CleaniCal^®^ and Calcipex II^®^ — which contain viscous vehicles — had significantly lower levels of bacteria and EPS compared with water-based Calasept Plus™, suggesting that they had higher efficacy in biofilm removal. Furthermore, CleaniCal^®^ removed more bacteria and EPS than Calcipex II^®^. In this respect, it was demonstrated that the NMP-based medicament, CleaniCal^®^, exerted the best dispelling effect on *E. faecalis* biofilms compared with other medicaments containing water or PG. These results also indicate that the structure of biofilms was dispersed and dispelled easily by irrigation.

Lastly, according to the CLSM results, the morphological features of the biofilms were investigated after treating the root canal wall with various medicaments. FE-SEM images showed the specimens treated with Calasept Plus™ were covered by biofilms, whereas the specimens treated with other medicaments were not (Figure 3a-d). Furthermore, the specimen treated with CleaniCal^®^ had a cleaner image than the one that had Calcipex II^®^ treatment, suggesting that CleaniCal^®^ was completely removed from the specimen. Biofilms are organic layers present on root canal walls in nature that consist of bacteria, EPS, proteins, and lipids. Therefore, it was speculated that the viscous vehicle, which is an organic solvent, might contribute to dispelling biofilms composed of organic substrates. A supplementary experiment was performed using nail polish to support this speculation. The experiment consists of a film-forming polymer that can be dissolved by an organic solvent. In fact, NMP is used for pain stripping, graffiti removal, and industrial cleanup. It has been approved by Food and Drug Administration (FDA) as a biodegradable solvent and is listed as generally recognized as safe (GRAS).^18^ In this respect, it was assumed that nail polish could model a biofilm and NMP could serve as the “stripper” intracanal medicament. As shown in Figure 3a’-d’, the nail polish immersed in NMP solution was completely removed from the bovine tooth surface. However, the nail polish was not removed in other groups. Lim, et al.^19^ (2017) demonstrated that CleaniCal^®^ was removed more effectively from the human root canal compared with ApexCal^®^ (Ivoclar Vivadent, Schaan, Liechtenstein), which contains PEG and Calcipex II^®^. They suggested the favorable effect might be due to the higher solubilizing efficiency of NMP compared with PEG or PG. Thus, the null hypothesis was rejected.

## Conclusions

Overall, CleaniCal^®^ performed better than Calasept Plus™ and Calcipex II^®^ in the removal efficacy of *E. faecalis* biofilms. The results also suggest that the effect might be due to the potent dissolving effect of NMP on organic substances. Within the limitations of this study, CleaniCal^®^ has the potential to be used as a recommended intracanal medicament.
